# Self-processing characteristics from first-person and third-person perspectives in individuals with social anxiety disorder: insights into negative bias

**DOI:** 10.3389/fpsyt.2023.1283624

**Published:** 2024-02-05

**Authors:** Huating Wu, Caizhen Yue, Fasheng Cao, Yihong Long, Yan Wang

**Affiliations:** ^1^College of National Culture and Cognitive Science, Guizhou Minzu University, Guiyang, China; ^2^School of Public Administration, South China University of Technology, Guangzhou, China; ^3^The Faculty of Education, Southwest University, Chongqing, China; ^4^International Affairs Office, Chongqing University of Arts and Sciences, Chongqing, China

**Keywords:** social anxiety disorder, self-processing characteristics, negative bias, first-person perspective, third-person perspective

## Abstract

**Background:**

As one of the most common psychological problems, social anxiety disorder (SAD) has lots of negative effects on the physical and mental development of individuals, such as decreasing the quality of interpersonal relationships, and even causing depression, suicidal ideation, etc., as well as leads individuals to generate mental illness stigma. The mental illness stigma that individuals perceive affects not only how they perceive themselves (first-person perspective) but also how they perceive others’ appraisals of them (third-person perspective), which further exacerbates their anxiety symptoms.

**Objective:**

The study aims to explore the self-processing characteristics of individuals with social anxiety disorder from the first-person perspective and the third-person perspective.

**Methods:**

This study adopted the self-referential paradigm to conduct the recognition memory test on individuals with social anxiety disorder (30 participants in experiment 1) and individuals without social anxiety disorder (31 participants in experiment 2) in the two experiments.

**Results:**

In experiment 1, the recognition rate of individuals with social anxiety disorder under the self-appraisals condition was significantly higher than that under the condition of appraisals on mothers; in the three conditions of self-appraisals, appraisals on mothers and mothers’ reflected appraisals, the recognition rate of negative trait adjectives was significantly higher than that of positive trait adjectives. In experiment 2, there was no significant difference in recognition rate of individuals without social anxiety disorder under the three conditions, and the recognition rate of positive trait adjectives was significantly higher than that of negative trait adjectives under the three conditions.

**Conclusion:**

Individuals with social anxiety disorder have a negative bias in self-processing and are more likely to focus on self-information, which is different from the self-positive bias of individuals without social anxiety disorder. This study can be beneficial to know the self-cognitive characteristics of individuals with social anxiety disorder, help them get rid of negative cognitive patterns, and remove the mental illness stigma.

## Introduction

As one of the most common psychological problems, the incidence of social anxiety disorder (SAD) is between 5 and 10% (1, 2). It refers to individuals’ fear of being paid excessive attention by others or being negatively appraised by others in social settings, resulting in obvious tension and fear (3). Individuals with SAD usually show impaired social function, fear of criticism, avoidance of social situations, and other behaviors, which are reflected in interpersonal relationship problems and relationship maintenance problems (4, 5). Individuals’ anxiety can also have a negative influence on their physical and mental health. Previous studies have found that SAD has a significant positive correlation with suicidal ideation (6), and individuals with SAD are more likely to maintain a high state of mental tension, resulting in extreme behaviors such as alcoholism, drug addiction, and even suicide (7).

### The mental illness stigma of individuals with social anxiety disorder

Social anxiety disorder not only causes many negative effects on individuals but also makes individuals produce mental illness stigma (8). The stigma is usually used as a sign or a label indicating that the marked person has negative attributes that are undesirable to society, resulting in the loss of other personal values and the degradation of social identity (9). On the one hand, because individuals with psychological problems such as social anxiety have some characteristics that are different from ordinary people, others often negatively regard or appraise individuals with such “stain” because of these humiliating characteristics (10). On the other hand, these special individuals often perceive mental illness stigma as a result of negative external appraisals (11, 12). Relevant studies have found that individuals with SAD can perceive mental illness stigma through self-perception (13). They are very sensitive to others’ appraisals, and even if others positively appraise individuals with SAD, they will be distorted into negative information to a large extent (14, 15), who regard themselves as “stigmatized” individuals. Moreover, individuals with SAD can perceive mental illness stigma through others’ appraisals (16). According to the theory of social interaction, everyone has an “me-in-the-situation,” i.e., appraising individual behaviors by understanding others’ reactions (17), while individuals with SAD know and cognize themselves through others’ appraisals, and they think themselves as “negative” as others’ appraisals, thus perceiving the stigma from the outside (18). The perception of self-identity stigma of individuals with SAD not only exacerbates their anxiety symptoms but also affects how they view themselves and how they view others’ appraisals of themselves (19, 20).

### Self-negative bias

As a special group, individuals with social anxiety disorder tend to have less positive bias than those without SAD. Although people often attribute positive events to internal and stable factors, this attribution tendency is difficult to be found in individuals with social anxiety disorder (21, 22). They make internal attributions to negative events, believing that failure is their lack of ability but success has nothing to do with them (23). Huppert et al. found that SAD causes individuals to pay excessive attention to negative information, and changes in SAD symptoms predict subsequent changes in negative cognition. Individuals with SAD will selectively focus on, interpret and memorize negative information in interpersonal situations, and have a negative interpretive bias (24). According to Beck’s cognitive theory, he believes that the schemas of individuals with SAD are usually negative. When external stimuli act, the schemas related to negative information in the brain are more easily activated, then individuals will selectively process and encode their perceived information, thus forming a negative cognitive processing bias (25). However, individuals without SAD generally show more positive biases (26, 27); that is, they usually tend to view themselves with a more positive attitude. For example, individuals usually perceive themselves as having more positive (and fewer negative) traits and abilities (28), and rate positive traits as self-related and negative traits as self-unrelated. Positive traits or outcomes are attributed to internal and stable personality traits (29), while negative traits are rated as unrelated to the individuals’ traits (30), and the responses to self-positive adjectives were faster than those to self-negative adjectives (31). Individuals with SAD show a different bias from those without SAD, and it is necessary to further discuss the stability of negative bias in individuals with SAD.

### Self-focused attention

In addition to the negative bias, individuals with social anxiety disorder also have self-focused attention (32). In the process of self-knowledge, individuals usually adopt both the first-person perspective (viewing themselves from their own perspective) and the third-person perspective (viewing themselves from others’ perspective) (33). While individuals with SAD more tend to view themselves from their own perspectives and tend to focus on self-related information (34). They begin their self-knowledge by collecting external information, such as the opinions of their mothers, classmates, and friends, but they attach more importance to appraising themselves from the perspective of the self (35). In social settings, they always deviate their attention from social situations, ignore external social information, shift their attention to themselves, and pay high attention to self-related information (36). Heimberg’s cognitive-behavioral model of SAD shows that when anxious individuals enter the public scenes, they will form self-mental representations, take themselves as the focus of cognition, and make self-appraisals based on the view of the “self” (37). However, excessive self-focused attention tends to lead to the ambiguity of self-concept and the fuzziness of self-appraisal (38). Individuals with SAD attach great importance to their own views in self-processing and will have biases in self-appraisals (39, 40).

### Hypotheses

This study explores the self-cognitive characteristics of individuals with social anxiety disorder by adopting the adapted self-reference paradigm from the first-person and the third-person perspectives. The self-reference effect will affect the connection between memory and perceptual stimuli and can better promote to process self-related information (41, 42). In addition, in the previous self-reference paradigm, the role of the mother is very important for Chinese individuals, and the Chinese self contains the component of the mother (43). Therefore, the role of the mother is chosen as one of the encoding conditions in this study. In the self-reference paradigm, the test procedure usually includes three stages: encoding, interference, and recognition. In the encoding phase, the participants were asked to rate the self-related information from the first-person perspective (or the third-person perspective), followed by interference through irrelevant tasks, and finally a recognition test. Individuals’ self-processing characteristics were explained by analyzing response times and recognition rates of trait adjectives judgment (44). Based on these, the study explored the self-processing characteristics of individuals with SAD through the comparison between individuals with SAD (experiment 1) and individuals without SAD (experiment 2). The researchers hypothesize that (1) compared with individuals without SAD, individuals with SAD have negative self-processing bias (2) and individuals with SAD tend to more focus on self-information.

## Experiment 1

### Purpose

Experiment 1 mainly explores the cognitive processing characteristics of individuals with social anxiety disorder under the first-person and the third-person perspectives. The participants were firstly asked to judge the trait adjectives of self-appraisals, mothers’ reflected appraisals, and appraisals on mothers in the learning stage, and then entered the recognition stage. Based on this, it is predicted that individuals with SAD show a negative bias and tend to pay more attention to self-appraisals in the learning stage and the recognition stage.

### Methods

#### Participants

This study used *f* = 0.27 as G-Power 3.1.9 [*ɑ* = 0.05; (45)] to measure the medium effect of the primary results and estimated the required sample size. The results indicated that the sample sizes required for experiment 1 and experiment 2, respectively, are 24 persons. Before the start of the experiments, the researchers screened 393 participants from Chongqing University of Arts and Sciences by using the IAS Social Anxiety Scale (Cronbach’s α coefficient is 0.8) compiled by Leary (46). Finally, 30 participants were selected to participate in experiment 1 (28 females, 2 males, mean age of 19.10 ± 1.2 years). Their original IAS mean score was 42.10, and the standard deviation was 6.82, indicating moderate anxiety. Experiment 1 was taken as the experimental group. All participants had no previous psychiatric history such as depression and other anxiety disorders, were familiar with computer operation, and had not participated in this type of experiment before. This study was performed in accordance with the recommendations of the Ethics Committee of Chongqing University of Arts and Sciences. The Ethics Committee of Chongqing University of Arts and Sciences approved the protocol. All the participants of the study provided written informed consent in accordance with the Declaration of Helsinki, and they received financial compensation of 10.00 RMB at the end of the study.

#### Experimental design

This study used the 3 (encoding condition: self-appraisals/mothers’ reflected appraisals/appraisals on mothers) × 2 (valence: positive/negative) within-subjects design, in which independent variable 1 was the processing task (self-appraisals/mothers’ reflected appraisals/appraisals on mothers) and independent variable 2 was trait adjectives judgment (positive/negative), and the dependent variables for the present experiment were the response times scores of trait adjectives judgment in the learning phase and the recognition rate scores in the recognition phase. SPSS22.0 software was used for data processing and analysis in the study.

#### Materials

From Dengfeng Wang’s (47) text version of *Rating on Desirability, Meaningfulness, Familiarity, and Modernity of 1,520 Chinese personality trait adjectives* in *Explorations of Chinese Personality*, 240 personality trait adjectives were selected, including 120 positive adjectives (e.g., optimistic and cheerful) and 120 negative adjectives (e.g., pessimistic and stupid). The 240 trait adjectives were divided into 6 groups, each group of 40 personality trait adjectives, and the valence, meaningfulness, and familiarity of each group were average, which were consistent with previously studied materials (48). Three groups of trait adjectives were randomly selected from the six groups to be judged in the learning stage, and the other three groups were judged as new items in the test stage.

#### Procedures

Before the experiment, the participants needed to do several exercises to help them understand the task requirements, and they did not enter the formal experiment until they were fully proficient in the experimental operation. The materials used in the practice experiments were not presented in the formal experiments. Then in the coding task, the participants were asked to make mothers’ reflected appraisals (e.g., “Does my mother think I’m a kind person?”), appraisals on mothers (e.g., “Is my mother kind?”), and self-appraisals (e.g., “Am I kind?”) for the reference processing. Then, in the interference phase, the participants joined in Raven’s Progressive Matrices to remove their attention from the coding task. Finally, in the recognition stage, the researchers mixed 120 trait adjectives that had not appeared in the formal experiment and 120 trait adjectives that had appeared in the formal experiment, and presented them randomly on the screen one by one, so that the participants could judge whether these trait adjectives had appeared in the previous stage.

### Results

#### Response times in the learning stage

The 3 (encoding condition: self-appraisals/mothers’ reflected appraisals/appraisals on mothers) × 2 (valence: positive/negative) repeated-measures ANOVA was conducted for the response times in the learning stage in experiment 1 (seen in Table 1), taking the participants’ response times in the learning stage as the dependent variable, taking encoding conditions and adjectives valence as the independent variables and as within-subjects factors. The results showed that the main effect of the encoding conditions was not significant [*F*(2,60) = 0.94, *p* > 0.05, $\eta_{p}^{2}$ = 0.03]. The main effect of PoS (parts of speech) was significant [*F*(1,30) = 5.04, *p* < 0.05, $\eta_{p}^{2}$ = 0.15], and the interaction between encoding conditions and PoS was not significant [*F*(2,60) = 0.72, *p* > 0.05, $\eta_{p}^{2}$ = 0.02]. The results showed that the participants’ response times to positive adjectives (*M* = 1639.04, *SD* = 91.36) were significantly longer than those of negative adjectives (*M* = 1588.27, *SD* = 87.49) under the three encoding conditions, *p* < 0.05.

**Table 1:** Mean and standard deviation of response times in the learning stage.

	Mean	Standard deviation
RT of SA to PA	1617.94	476.56
RT of SA to NA	1519.83	502.19
RT of MRA to PA	1651.68	514.36
RT of MRA to NA	1623.14	483.76
RT of AM to PA	1647.51	611.98
RT of MA to NA	1621.83	592.68

RT, response times; PA, positive adjectives; NA, negative adjectives; SA, self-appraisals; MRA, mothers’ reflected appraisals; AM, appraisals on mothers.

#### Recognition rates in the recognition stage

Taking the participants’ recognition rates in the recognition stage as the dependent variable, the 3 (encoding conditions: self-appraisals, appraisals on mothers, mothers’ reflected appraisals) × 2 (PoS: positive, negative) repeated-measures ANOVA was performed (seen in Table 2). The results showed as follows: the main effect of the encoding conditions was significant [*F*(2,60) = 6.03, *p* < 0.05, $\eta_{p}^{2}$ = 0.17]; the main effect of PoS was significant [*F*(1,30) = 6.52, *p* < 0.05, $\eta_{p}^{2}$ = 0.18]; and the interaction between the encoding conditions and PoS was not significant [*F*(2, 60) = 2.32, *p* > 0.05, $\eta_{p}^{2}$ = 0.07]. The results of the post-hoc test analysis found that the recognition rate of the participants’ self-appraisals (*M* = 0.39, *SD* = 0.01) was significantly higher than that of appraisals on mothers (*M* = 0.35, *SD* = 0.01), *p* < 0.05. Under the three encoding conditions, the recognition rate of negative adjectives from the participants (*M* = 0.39, *SD* = 0.01) was significantly higher than that of positive adjectives (*M* = 0.36, *SD* = 0.01), *p* < 0.05.

**Table 2:** Mean and standard deviation of recognition rates in the recognition stage.

	Mean	Standard deviation
RR of SA on PA	0.38	0.07
RR of SA on NA	0.40	0.06
RR of MRA on PA	0.36	0.06
RR of MRA on NA	0.37	0.07
RR of AM on PA	0.33	0.07
RR of AM on NA	0.38	0.08

RR, recognition rate; PA, positive adjectives; NA, negative adjectives; SA, self-appraisals; MRA, mothers’ reflected appraisals; AM, appraisals on mothers.

### Discussion

The self-reference paradigm was used to explore the self-processing bias of individuals with social anxiety disorder in experiment 1. The results showed that the participants’ response times to positive trait adjectives judgment were significantly longer than those to negative trait adjectives judgment in the learning stage, but there were no significant differences among the main effects of encoding conditions (self-appraisals, mothers’ reflected appraisals, and appraisals on mothers). In the recognition stage, the participants’ recognition rates of negative trait adjectives judgment were significantly higher than those of positive trait adjectives judgment, and the recognition rates of self-appraisals were significantly higher than those of appraisals on mothers in the three encoding conditions. The results indicated that individuals with SAD have a negative self-processing bias; compared with others’ appraisals, individuals with SAD more focus on self-appraisals and self-related information.

## Experiment 2

### Purpose

Experiment 2 aims to probe into the self-processing characteristics of individuals without social anxiety disorder from the first-person and the third-person perspectives. The self-processing characteristics of individuals with SAD were explored by comparing the group with SAD (experiment 1) with the group without SAD (experiment 2).

### Methods

#### Participants

Experiment 2 randomly selected 31 individuals (16 males, 15 females, mean age of 19.58 ± 1.2 years) without social anxiety disorder from Chongqing University of Arts and Sciences, whose average IAS score was lower than 20 (11.93 ± 4.79) (46). Participants had normal or corrected vision and no history of neurological disorders. This study was performed in accordance with the recommendations of the Ethics Committee of Chongqing University of Arts and Sciences. All participants of the study provided written informed consent in accordance with the Declaration of Helsinki, which was approved by the Ethics Committee of Chongqing University of Arts and Sciences, and they received a financial compensation of 10.00 RMB at the end of their studies. The essential difference between experiment 1 and experiment 2 is whether the participants suffer from SAD, which means that all participants with SAD were included in experiment 1 as the experimental group, and the participants without SAD were included in experiment 2 as the control group.

#### Materials and procedures

The experimental design, materials, and procedures for experiment 2 were the same as for experiment 1, both taking 240 personality trait adjectives to create a stimuli list in the encoding and recognition phases and encoding adjectives in the same way. The difference is that experiment 2 selected participants without social anxiety disorder.

### Results

#### Response times in the learning stage

Taking the participants’ response times in the learning stage as the dependent variable, the 3 (encoding conditions: self-appraisals, mothers’ reflected appraisals, appraisals on mothers) × 2 (PoS: positive, negative) repeated-measures ANOVA was performed (seen in Table 3). The results indicated that the main effect of the encoding conditions was significant [*F* = (2,62) = 3.36, *p* < 0.05, $\eta_{p}^{2}$ = 0.10], while the main effect of PoS was not significant [*F* = (1,31) = 0.22, *p* > 0.05, $\eta_{p}^{2}$ = 0.007]. The interaction of the encoding conditions and PoS was marginally significant [*F* = (2,62) = 3.80, *p* < 0.05, $\eta_{p}^{2}$ = 0.11]. The analyses from post-hoc tests showed that the response times of mothers’ reflected appraisals (*M* = 1880.55, *SD* = 98.99) were significantly longer than those of self-appraisals (*M* = 1743.99, *SD* = 101.26), *p* < 0.05. Repeated-measures ANOVA indicated that the response times to negative adjectives (*M* = 1944.40, *SD* = 111.24) were significantly longer than those of positive adjectives (*M* = 1816.70, *SD* = 94.90), *p* < 0.05, under the condition of mothers’ reflected appraisals.

**Table 3:** Mean and standard deviation of response times in the learning stage.

	Mean	Standard deviation
RT of SA to PA	1768.64	588.81
RT of SA to NA	1719.34	578.97
RT of MRA to PA	1816.70	528.42
RT of MRA to NA	1944.40	619.37
RT of AM to PA	1735.74	600.67
RT of MA to NA	1717.52	535.04

RT, response times; PA, positive adjectives; NA, negative adjectives; SA, self-appraisals; MRA, mothers’ reflected appraisals; AM, appraisals on mothers.

#### Recognition rates in the recognition stage

Taking the participants’ recognition rates in the recognition stage as the dependent variable, 3 (encoding conditions: self-appraisals, mothers’ reflected appraisals, appraisals on mothers) × 2 (PoS: positive, negative) repeated-measures ANOVA was performed (seen in Table 4). The results showed that the main effect of the encoding conditions was not significant [*F* = (2,62) = 1.31, *p* > 0.05, $\eta_{p}^{2}$ = 0.04], the main effect of PoS was significant [*F* = (1,31) = 5.25, *p* < 0.05, $\eta_{p}^{2}$ = 0.14], and there was no significant interaction between the encoding conditions and PoS [*F* = (2,62) = 1.45, *p* > 0.05, $\eta_{p}^{2}$ = 0.37]. The participants’ recognition rates on positive adjectives (*M* = 0.36, *SD* = 0.01) were significantly higher than those on negative adjectives (*M* = 0.33, *SD* = 0.01) under the three encoding conditions, *p* < 0.05.

**Table 4:** Mean and standard deviation of recognition rates in the recognition stage.

	Mean	Standard deviation
RR of SA on PA	0.37	0.08
RR of SA on NA	0.33	0.07
RR of MRA on PA	0.36	0.08
RR of MRA on NA	0.34	0.09
RR of AM on PA	0.35	0.08
RR of AM on NA	0.33	0.09

RR, recognition rate; PA, positive adjectives; NA, negative adjectives; SA, self-appraisals; MRA, mothers’ reflected appraisals; AM, appraisals on mothers.

#### Comparison of results of the experimental group and the control group

The researchers compared the results of experiment 1 and experiment 2 to explore the cognitive bias of self-processing in individuals with social anxiety disorder from different perspectives. The 2 (experiments: 1/2) × 3 (encoding conditions: self-appraisals, mothers’ reflected appraisals, appraisals on mothers) × 2 (valence: positive/negative) repeated-measures ANOVA was adopted to, respectively, measure response times and recognition rates. The results showed that there was no interaction among the three variables (experiments, encoding conditions, and valence) in the learning stage. In the recognition stage, the interaction of encoding conditions × experiments was not significant [*F* = (2,122) = 1.55, *p* > 0.05, $\eta_{p}^{2}$ = 0.26], there was no significant difference [*F* = (2,122) = 1.22, *p* > 0.05, $\eta_{p}^{2}$ = 0.02] in the interaction of encoding conditions × valence × experiments, but there were significant differences in the interaction of valence × experiments [*F* = (2,122) = 11.71, *p* < 0.05, $\eta_{p}^{2}$ = 0.17]. The results from post-hoc tests showed that the recognition rates of negative adjectives (*M* = 0.39, *SD* = 0.01) were significantly higher than those of positive adjectives (*M* = 0.36, *SD* = 0.01) in experiment 1, but the participants’ recognition rates of positive adjectives (*M* = 0.36, *SD* = 0.01) were significantly higher than those of negative adjectives (*M* = 0.33, *SD* = 0.01) in experiment 2. The results indicated that individuals with SAD had a negative processing bias in the process of self-processing, compared with those without SAD.

### Discussion

Experiment 2 used the self-reference paradigm to explore the self-cognitive bias of individuals without social anxiety disorder. The results showed that the recognition rates of positive trait adjectives judgment were significantly higher than those of negative trait adjectives judgment in the recognition stage. By comparing the results of experiment 1 and experiment 2, it is found that individuals with SAD have a significant negative processing bias, compared with those without SAD.

## General discussion

This study aimed to explore the self-cognitive characteristics of individuals with social anxiety disorder under the first-person and the third-person perspectives through the self-referential paradigm. The results indicated that there were differences in cognitive processing between individuals with SAD and those without SAD. Different from the self-positive bias of individuals without SAD, individuals with social anxiety showed a negative self-processing bias, and individuals with SAD had significantly higher recognition rates of self-appraisals than those of appraisals on mothers, indicating that individuals with SAD are more inclined to pay attention to self-information. The participants of the study were college students with moderate SAD (mean age of 19.58 ± 1.2 years). The results of this study can be further extended to other groups and help to better understand the cognitive characteristics of college students with moderate SAD.

In this study, individuals with SAD had significantly higher recognition rates for negative trait adjectives than those for positive trait adjectives, while individuals without SAD had significantly higher recognition rates for positive trait adjectives than those for negative trait adjectives. This indicates that compared with individuals without SAD, individuals with SAD have a significantly negative processing bias in self-processing. Previous studies have found that individuals with SAD have a negative interpretation bias toward external things; that is, they tend to interpret ambiguous situations in a negative way (49). According to the theory of the cognitive model of social phobia, individuals with SAD have irrational beliefs about negative information, and they are more likely to have relatively negative emotional experiences than others (50). They often rely on negative cues when recognizing or judging external things, which leads to negative cognitive bias (51). In addition, the study by Crestani et al. found that patients with anxiety disorders had a loss of GABA receptors in the hippocampus and the base of the frontal brain region, which impaired their explicit memory tendency for threatening events and led to an increase in negative associations (52). Individuals with SAD tend to remember negative information in social situations and allocate limited attention to anxiety-generating events and distorted self-images (53). Excessive attention to negative information in the social process leads to unbalanced distribution of attention resources, thus interfering with individuals’ objective appraisals of the social environment and forming a negative self-processing bias (54). In addition, the negative characteristics of SAD will cause individuals to have mental illness stigma, and the stigma perceived by individuals will trigger their fear of social exclusion and slander, resulting in increased pressure and self-cognitive dissonance, which will aggravate the symptoms of SAD (55).

This study found that the recognition rate score of individuals with social anxiety disorder was significantly higher than that of appraisals on mothers in experiment 1, while there was no significant difference in the main effect in the recognition rate stage of individuals without SAD in experiment 2. This suggests that individuals with SAD are more likely to pay attention to self-related information than those without SAD. The results of this study are consistent with previous studies, but the difference is that this study explores the cognitive characteristics of individuals with SAD from the third-person perspective. The basic feature of individuals with SAD is their high attention to self-information (56). Woody conducted an experiment on 20 individuals with SAD and 20 individuals without SAD through questionnaires and interviews and found that the participants with SAD paid more attention to self-appraisals, and they often conducted self-construal from the viewpoint of “self” (57). Mansell et al. conducted an experiment with 64 college students by using the computerized reaction-time paradigm and found that individuals with SAD attach more importance to their internal feelings when they are faced with social threats (public speaking, communication, etc.) (58). Neuroscientific evidence also showed that Individuals with SAD show significant activation effects in the prefrontal cortex and amygdala when reading self-related information, but no significant activation effects when reading appraisals about others (59). On the one hand, individuals with SAD pay excessive attention to self-information, which leads them to allocate more attention to themselves in social situations, and more focus on their internal feelings, thus producing more anxiety experiences such as tension and fear. On the other hand, they pay more attention to themselves, leading their attention to a deviation from objective social situations, ignoring external social information, subjectively making poor self-performance judgment through internal body feelings, etc., resulting in a higher level of anxiety, interfering with the correct judgment of the external environment, and affecting their self-regulation. As a result, their social functioning is severely affected and they suffer greater mental distress (60).

Based on the findings of this study, it is important to provide positive guidance to individuals with social anxiety disorder. On the one hand, it is necessary to try to change the negative self-bias of individuals with SAD and form a positive self-schema. For example, it is necessary to guide them to form positive psychological suggestions. It is meaningful to let them learn to ignore negative information, attribute the cause of failure to changeable factors, often make positive self-appraisals, and avoid using negative words, etc., so as to help them change their emotions and behaviors accordingly (61). The causes of forming social anxiety can also be influenced by social relationships (such as others’ appraisals), which reflect the common views and consistent reactions of social members to individuals with SAD (14, 62). Therefore, society should actively focus on individuals with SAD and provide more positive feedback to them. On the other hand, individuals with SAD need to get rid of the self-focused tendency. They should be positively guided to view problems from the others’ perspectives and try to reduce their self-focused attention by shifting their focus of attention to the outside, observing changes in people or things around them, and searching for the best state in which they feel comfortable.

The study explores the self-processing characteristics of individuals with social anxiety disorder from the first-person and the third-person perspectives, respectively, which is conducive to understanding the cognitive characteristics of individuals with SAD and expanding the research perspective of self-bias of individuals with SAD. However, there are also some problems in this study. First, the sample size of this study is small. Although the study meets the requirement of a minimum number of each experiment according to the calculation of the sample size, the sample size is still small. Second, although the Cronbach’s α coefficient of the scale used in this study is satisfactory, it is not very high. LSAS Social Anxiety Scale and other scales with higher reliability can be used in future. These provide reference significance for future research studies. In future studies, the following aspects can be considered: first, in the self-referential paradigm, the mother condition in the experimental task can be changed to other important people (such as good friends or lovers) to further explore the stability of the results; second, different scientific paradigms, such as neuroscientific paradigms, can be used in future studies to deeply analyze the neural basis and cognitive characteristics of self-processing characteristics of individuals with SAD. Third, future studies can extend the research object to other groups, such as adolescents. Because adolescents are in a special state of psychological development, their emotions are easy to fluctuate, and their personalities are unstable, the cognitive characteristics of adolescents with SAD and the differences between them and other groups can be further explored.

In conclusion, the findings suggest that individuals with social anxiety disorder show stable negative bias and pay more attention to self-information from both the first-person and the third-person perspectives, which indicates that they more focus on the negative self-related information in the social environment and pay more attention to their inner feelings. Therefore, this study emphasizes the importance of giving positive social support to individuals with SAD and helping them to reduce self-focused attention.

## Data availability statement

The original contributions presented in the study are included in the article/supplementary material, further inquiries can be directed to the corresponding author.

## Ethics statement

The studies involving humans were approved by the Ethics Committee of Chongqing University of Arts and Sciences. The studies were conducted in accordance with the local legislation and institutional requirements. The participants provided their written informed consent to participate in this study.

## Author contributions

HW and CY constructed this study, drafted the initial manuscript, and designed the experiments. CY, FC, YL, and YW conducted all the experiments in the study. HW, CY, and FC processed and analyzed all the sequencing data. All the authors checked and revised the data and the manuscript. The final manuscript was approved for submission by all the authors who were agreeable to be responsible for this study.
